# Predicting the outcomes of pulmonary hypertension is a breathtaking task

**DOI:** 10.1007/s12471-020-01512-z

**Published:** 2020-10-28

**Authors:** M. A. M. Beijk, R. J. de Winter

**Affiliations:** Department of Cardiology, Heart Center, Amsterdam UMC, University of Amsterdam, Amsterdam Cardiovascular Sciences, Amsterdam, The Netherlands

Pulmonary hypertension (PH) is a pathophysiological disorder that may involve multiple clinical conditions and can complicate cardiovascular and respiratory diseases [1]. Despite treatment advances in recent years, PH remains a life-shortening condition. The clinical classification of PH categorises multiple clinical conditions into five groups according to their similar clinical presentation, pathological findings, haemodynamic characteristics and treatment strategy [2].

When PH is suspected based on symptoms, physical examination and echocardiography, a diagnostic algorithm can be used to identify the underlying pathophysiological disorder, as proposed in the European Society of Cardiology guidelines for the diagnosis and treatment of PH [1]. A comprehensive set of investigations can be used, such as electrocardiography, pulmonary function testing and arterial blood gas analysis, ventilation/perfusion lung scanning, computerised tomography (CT) imaging, cardiac magnetic resonance imaging, blood tests and genetic testing. Eventually, right heart catheterisation is required to confirm the diagnosis of pulmonary arterial hypertension (PAH) or chronic thromboembolic pulmonary hypertension (CTEPH).

PH is defined as an increase in mean pulmonary arterial pressure ≥25 mm Hg at rest as assessed by right heart catheterisation [3]. For adult patients with congenital heart disease, the cut-off value for PH has been reduced to >20 mm Hg in the recently published guidelines [4]. Especially in case of PAH, it is important to classify the patient’s risk of clinical worsening or death as low, intermediate or high, as this will guide medical treatment. The following parameters are used for risk assessment in PAH: (a) clinical signs of right heart failure, (b) progression of symptoms, (c) syncope, (d) World Health Organization (WHO) functional class, (e) 6-minute walking distance, (f) cardiopulmonary exercise testing, (g) *N*-terminal pro-brain natriuretic peptide (NT-proBNP) plasma levels, (h) imaging (echocardiography, cardiac magnetic resonance imaging), and (i) invasive haemodynamics [1]. The overall treatment goal in patients with PAH is to achieve a low-risk status, which is associated with a good exercise capacity, good quality of life, good right ventricular function and low mortality risk. Currently, there is no composite risk score calculator available for CTEPH to predict outcome or to guide treatment.

In this issue of the *Netherlands Heart Journal*, Duijnhouwer and colleagues report the outcome of PH and its association with pulmonary artery (PA) dilatation [5]. In this retrospective single-centre study, the authors tested the hypothesis that the PA diameter is associated with the disease stage, and thus with the prognosis, in 217 patients with PAH or CTEPH. Patients were included when right heart catheterisation confirmed the presence of PH, and when a thoracic CT scan and data on the New York Heart Association class, NT-proBNP level and 6‑minute walking distance were available. Although CT imaging was performed for different reasons and therefore not all scans included administration of contrast medium, the main PA diameter was measured in a standardised manner, i.e. perpendicular to the PA wall in a transversal view at the level of the aorta at which the right PA was also visible. For the purpose of the study, PA dilatation was defined as a diameter ≥29 mm for men and ≥27 mm for women, following the upper limit of normal (90th percentile) proposed by the Framingham Heart study. The primary endpoint was mortality, which was obtained from the municipal personal records database of the Netherlands. Even though known predictors of survival were confirmed, PA diameter at diagnosis was not associated with survival in PAH or CTEPH patients.

Duijnhouwer and colleagues should be complimented for their search for an easy to obtain, noninvasive parameter that may predict outcome in patients with PH. However, PH can occur in multiple and often complex clinical conditions, and it may be far-stretched to think that one size or one measurement should fit all. For example, the PA diameter may be enlarged in the absence of PH. An increase in PA size over time can be a feature of PH but is not necessarily an indication of increasing PA pressure; therefore, the correlation of PH with mean pulmonary arterial pressure is weak [6].

Moreover, within PH subgroups, different vascular and cardiac configurations are observed with CT, reflecting the heterogeneity of PH. In a systematic CT evaluation of 292 treatment-naive PAH patients from the ASPIRE Registry, the prevalence and prognostic value of CT pulmonary angiographic measurements were evaluated [7]. Multivariate analysis including vascular, cardiac, parenchymal and mediastinal changes revealed that only the size of the inferior vena cava area and the presence of pleural effusion or septal lines predicted outcome.

Recently, Swift, et al. build a diagnostic CT model and tested its prognostic significance [8]. The model incorporated main PA diameter, right ventricular outflow tract thickness, left ventricular area and interventricular septal angle [score  = −14.299 + (0.192 × main PA diameter, mm) + (0.518 × right ventricular outflow tract thickness, mm) − (0.001 × left ventricular area, mm^2^) + (0.068 × interventricular septal angle, degrees)]. The prognostic value of the CT model was assessed using a Kaplan-Meier analysis, with a mean follow-up time of 42 months; a threshold of ≥2.5 units was strongly predictive of mortality (*p* < 0.01).

Duijnhouwer et al. included four patient groups, each with a different disease course, prognosis and treatment modality. Based on the baseline characteristics of the study, the majority of the patients should have received guideline-recommended therapy. In idiopathic PAH, only a small number of patients respond to acute vasoreactivity testing and can be treated with high-dose calcium blockers. Patients with idiopathic PAH who do not show an adequate response and patients with PH associated with connective tissue disease in WHO functional class II or III have a class 1 recommendation to specific PH drug therapy, such as treatment with an endothelin receptor antagonist, a phosphodiesterase type 5 inhibitor, a guanylate cyclase stimulator, a prostacyclin analogue or a selective prostacyclin (IP) receptor agonist [1]. In patients with PH associated with congenital heart disease, treatment depends on the underlying cardiac malformations. Finally, in CTEPH patients who are technically operable, pulmonary endarterectomy is the treatment of choice, while balloon pulmonary angioplasty or medical treatment with a guanylate cyclase stimulator should be considered for technically inoperable CTEPH patients.

The lack of information on treatment and its potential interference with PA diameter, combined with the lack of multiple CT measurements over time, make it difficult to interpret the results of the study presented. The field of clinical PH research is challenging and may require a deep breath and a lot of patience.
